# Shiga Toxin 2a Induces NETosis via NOX-Dependent Pathway

**DOI:** 10.3390/biomedicines9121807

**Published:** 2021-12-01

**Authors:** Wouter J. C. Feitz, Samuel Suntharalingham, Meraj Khan, Carolina G. Ortiz-Sandoval, Nades Palaniyar, Lambert P. van den Heuvel, Nicole C. A. J. van de Kar, Christoph Licht

**Affiliations:** 1 Department of Pediatric Nephrology, Amalia Children’s Hospital, Radboud Institute for Molecular Life Sciences, Radboudumc, 6525 GA Nijmegen, The Netherlands; wouter.feitz@radboudumc.nl (W.J.C.F.); bert.vandenheuvel@radboudumc.nl (L.P.v.d.H.); nicole.vandekar@radboudumc.nl (N.C.A.J.v.d.K.); 2 Cell Biology Program, The Hospital for Sick Children Research Institute, Toronto, ON M5G 1X8, Canada; samuel.elijah.suntharalingham@gmail.com (S.S.); carolina.ortiz@sickkids.ca (C.G.O.-S.); 3 Program in Translational Medicine, The Hospital for Sick Children Research Institute, Toronto, ON M5G 1X8, Canada; meraj.khan@sickkids.ca (M.K.); nades.palaniyar@sickkids.ca (N.P.); 4 Department of Laboratory Medicine and Pathobiology, Institute of Medical Sciences, University of Toronto, Toronto, ON M5S 1A8, Canada; 5 Department of Pediatric Nephrology, Academic Hospitals Leuven, 3000 Leuven, Belgium; 6 Department of Development and Regeneration, Academic Hospitals Leuven, 3000 Leuven, Belgium; 7 Division of Nephrology, The Hospital for Sick Children, Toronto, ON M5G 1X8, Canada; 8 Department of Pediatrics, University of Toronto, Toronto, ON M5S 1A8, Canada

**Keywords:** hemolytic uremic syndrome, shiga toxin, STEC-HUS, neutrophil extracellular traps, NADPH-oxidase-dependent pathway

## Abstract

Shiga toxin (Stx)-producing *Escherichia coli* (STEC) infection is the most common cause of hemolytic uremic syndrome (HUS), one of the main causes of acute kidney injury in children. Stx plays an important role in endothelium damage and pathogenesis of STEC-HUS. However, the effects of Stx on neutrophils and neutrophil extracellular trap (NET) formation are not well understood. In this study, we investigated how Stx2a affects NET formation and NETotic pathways (NADPH or NOX-dependent and -independent) using neutrophils isolated from healthy donors and patients with STEC-HUS, during the acute and recovery phase of the disease. Stx2a dose-dependently induced NETosis in neutrophils isolated from both healthy controls and STEC-HUS patients. NETosis kinetics and mechanistic data with pathway-specific inhibitors including diphenyleneiodonium (DPI)-, ERK-, and P38-inhibitors showed that Stx2a-induced NETosis via the NOX-dependent pathway. Neutrophils from STEC-HUS patients in the acute phase showed less ROS and NETs formation compared to neutrophils of the recovery phase of the disease and in healthy controls. NETs induced by Stx2a may lead to the activation of endothelial cells, which might contribute to the manifestation of thrombotic microangiopathy in STEC-HUS.

## 1. Introduction

Hemolytic uremic syndrome (HUS) is a thrombotic microangiopathy (TMA) primarily characterized by hemolytic anemia, thrombocytopenia, and acute renal failure [1]. HUS predominantly affects children under the age of 5 years [2]. The majority of pediatric cases is triggered by an infection with Shiga toxin (Stx)-producing *Escherichia coli* (STEC-HUS), typically caused by ingestion of contaminated food [3]. There is no specific treatment for STEC-HUS. Patients are treated with supportive measures including renal replacement therapy, antihypertensive drugs, red blood cell transfusions, and fluid and electrolyte management [4]. A total of 70% of patients recover with time and supportive therapy [5], and mortality of STEC-HUS ranges from 2–5% [3].

Stx was considered to mainly affect the endothelium [6], but recent studies indicate that Stx also affects neutrophils [7,8,9,10]. Neutrophils belong to the innate immune system and play an essential role in the protection of the body against infections [11]. A high neutrophil blood count was correlated with a poor prognostic outcome in patients with STEC-HUS [2,5,12]. Multiple research groups have shown that the interaction of Stx with neutrophils leads to neutrophil activation with a local inflammatory response, activation, and damage of the endothelium as a result [8,10]. One of the abilities of neutrophils is the release of neutrophil extracellular traps (NETs), so called NETosis [13,14,15,16]. NET formation or NETosis generates a meshwork of nucleic fibers consisting of DNA and histones with embedded granular proteins to trap, disarm and kill pathogens extracellularly [13]. However, excess NETs may lead to some deleterious effects in surrounding tissues. Hence, understanding how Stx regulates NETosis is important.

NETosis can be divided into two subgroups, based on the involvement of NADPH-Oxidase (NOX) [17]. In NOX-dependent NETosis, certain agonists (e.g., PMA, LPS or bacteria activate the NOX-mediated ROS generation, leading to the activation of a set of kinases and degranulation of granular proteins (e.g., MPO, elastase). These kinases trigger transcription factors and induce chromatin decondensation [17]. By contrast, during NOX-independent NETosis, certain agonists (e.g., calcium ionophores such as A23187 or ionomycin) induce mitochondrial ROS [17]. Calcium influx also activates peptidylargininedeiminase 4 (PAD4) and helps to translocate the enzyme into the nucleus; PAD4 citrullinates the histone 3 (CitH3). In this pathway, different sets of kinases and transcription factors are involved along with citrullination to decondense the chromatin.

Although it was shown that neutrophils release NETs after direct stimulation with Stx [10], the underlying pathways involved are still unknown. The main aim of this study is the investigation of different pathways involved in NETosis caused by this toxin. Stx2a was used for experiments in this study because it is one of the most common subtypes of Stx and related with a more severe type of disease [18]. We also studied different pathways in neutrophils derived from healthy donors and patients in the acute and recovery phase of STEC-HUS.

## 2. Materials and Methods

### 2.1. Ethics

This study was approved by The Hospital for Sick Children Research Ethics Board, Toronto, ON, Canada (REB, Number 1.000.039.544). Written informed consent was obtained with a signature from healthy donors and all STEC-HUS patients or parents/legal guardians of patients whose neutrophils were isolated for this study. This study was executed in keeping with the regulations of the Declaration of Helsinki.

### 2.2. Human Peripheral Blood Polymorphonuclear Neutrophils

Polymorphonuclear neutrophils were isolated from the peripheral blood of healthy donors and STEC-HUS patients (*n* = 3) in the acute phase and after 3–5 months of recovery using K2-EDTA blood collection tubes (Becton, Dickinson and Co., Franklin Lakes, NJ, USA). Neutrophils were purified from peripheral blood with the use of PolymorphPrepTM (Axis-Shield, Oslo, Norway) following manufacturer’s protocol. Blood was layered over PolymorphPrepTM solution in a 1:1 ratio and was centrifuged at 600×*g* for 35 min with no brake or acceleration. Next, the polymorphonuclear neutrophil layer was collected and washed with 0.425% (*w/v*) NaCl with 10 mM 4-(2-hydroxyethyl)-1-piperazineethanesulfonic acid (HEPES) (Gibco, Thermo Fisher, Waltham, MA, USA) to eliminate residues of PolymorphPrepTM. A hypotonic solution of 0.2% (*w/v*) NaCl or NH4-ACK buffer was used twice for 30 s to get rid of residual red blood cells. To obtain isotonic conditions, neutrophils were mixed with an equal volume of 1.6% (*w/v*) NaCl solution with 20 mM HEPES buffer. Cells were washed till no red blood cell debris or soluble components were left. Neutrophils were resuspended in RPMI 1640 medium (Invitrogen, Carlsbad, CA, USA) supplemented with 10 mM HEPES buffer. A hemocytometer combined with trypan blue staining was used for quantification of cell density and determining the viability. Further, cytospin preparations were used to estimate the purity of isolated neutrophils. Neutrophil preparations with >95–98% purity was used for the experiments.

### 2.3. Shiga Toxin Type 2a

Stx2a was ordered from Phoenix Lab (Tufts Medical Center, Boston, MA, USA). The amount of LPS remaining in these preparations was determined to be less than 0.10 U/mL.

### 2.4. SYTOX Green NETosis Assay

To estimate NETosis kinetics of neutrophils, a SYTOX green fluorescence assay was used (SytoxGreen is an impermeable chromatin binding dye, signal shows the real time release of DNA in activated neutrophils either by media control or agonists), as we have previously done [19,20]. A concentration of 5 µM SYTOX green (Invitrogen, Thermo Fisher, MA) in 100 µL media containing 50,000 neutrophils was added. This was seeded into a 96-well plate (Sarsted, Numbrecht, Germany). Stx2a in a concentration of 0.01–1.0 µg/mL, 25–100 nM of phorbol myristate acetate (PMA) (Sigma-Aldrich, Saint Louis, MO, USA), 5–20 µg/mL of LPS derived from *E. coli* 0218 (Sigma-Aldrich, Saint Louis, MO), 2–8 µM of A23187 (Sigma-Aldrich, Saint Louis, MO) or 5–20 µM of ionomycin (Sigma-Aldrich, Saint Louis, MO, USA) was added and placed in a 37 °C incubator with 5% CO_2_. SYTOX green fluorescence intensity was measured every 30 min for 4–8 h with the use of a fluorescence plate reader at 504 nm excitation and 523 nm emission. To compare condition to condition and day to day experimental outcome, raw data were normalized in two steps. Firstly, data obtained at baseline (zero time point) were subtracted from other time points of the same condition to reduce the background. Secondly, subtracted background data at each time point was divided by the respective fluorescence data obtained with 0.5% Triton condition. Triton treatment resulting in cell lysis and release of the entire DNA content determined maximal fluorescence (SytoxGreen) and served as positive control. NETotic Index as denoted as % of maximum DNA release as previously published. Experiments were done in triplicates and quadruplicates. For all conditions involving NETosis inhibitors, cells were preincubated with either the NOX inhibitor diphenyleneiodonium (DPI) (20 μM), ERK inhibitor FR180204 (20 µM), p38 inhibitor SB20190 (2.0 µM) or the general ROS scavenger N-acetyl-l-cysteine (NAC) (5 mM). These inhibitors including DPI (cat# D2926), FR180204 (cat# SML0320), SB20190 (cat# S7067) and NAC (cat# A9165-5G) were bought from Sigma-Aldrich, Saint Louis, MO, USA.

### 2.5. Immunofluorescence Staining and Imaging

A total of 100,000 neutrophils in 100 µL media per well was seeded in a 12-well chamber slide (BD Falcon, Thermo Fisher, MA, USA). Cells were fixed with 4% Paraformaldehyde (PFA) (Electron Microscopy Sciences, Pennsylvania) in Phosphate Buffered Saline (PBS) (Wisent Bioproducts, QC, Canada) and stored overnight at 4 °C. Wells were washed 3 times with 1X PBS. 0.05% Triton was added and stored for 15 min to permeabilize cells. Cells were blocked with 4% bovine serum albumin (BSA) (Sigma-Aldrich, St. Louis, MO, USA) in PBS for 1 h. After 1 h, human anti-CD11b conjugated with Phycoerythrin (Biolegend, San Diego, CA, USA) in 1% BSA/PBS dilution 1:300, primary mouse antimyeloperoxidase (MPO) (Abcam, Cambridge, UK) in 1% BSA/PBS dilution 1:1000 and/or primary rabbit anti-citrullinated histone-3 (Abcam, Cambridge, UK) in 1% BSA/PBS dilution 1:500 was added and stored for 1 h at room temperature. After 1 h, corresponding species-specific antibodies (Thermo Fisher Scientific, Waltham, UK) in a dilution of 1:500 and DAPI (Invitrogen, Carlsbad, CA, USA) in a dilution of 1:100 were added and stored for 45 min at room temperature protected from light. Images were recorded with the use of an Olympus IX81 (Olympus corporation, Tokyo, Japan) inverted fluorescence microscope or with the use of Zeiss LSM 880 Airy scan (Carl Zeiss, Oberkochen, Germany) under control of Volocity software (PerkinElmer, Groningen, The Netherlands). For all conditions involving NETosis inhibitors, cells were preincubated either with DPI (20 μM), ERK inhibitor FR180204 (20 µM), p38 inhibitor SB20190 (2.0 µM) or general ROS scavenger N-acetylcysteine (NAC) (5 mM).

### 2.6. General Reactive Oxygen Species Detection Assay

Levels of general reactive oxygen species (ROS) were measured with dihydrorhodamine 123 (DHR123) (Life Technologies, Thermo Fisher, MA, USA). Neutrophils were incubated with 20 uM of DHR123 in PBS prior to seeding in a 96-well plate (Greiner, Biosigma, Italy). Neutrophils were incubated with Stx2a in a concentration of 0.01–1.0 ug/mL, or 25 nM PMA, 5 µg/mL LPS derived from *E. coli* 0218, 5 µM ionomycin or 4 µM A23187. Immediately after adding the agonists, the levels of ROS were measured every 10 min for 30 min, after 60 and after 90 min with the use of a Tunable Versa Max microplate reader (Molecular Devices, San Jose, CA, USA). Experiments were done in duplicates, triplicates or quadruplicates depending on the number of neutrophils collected from the donor. For all conditions involving NETosis inhibitors, cells were preincubated either with DPI (20 μM), ERK inhibitor FR180204 (20 µM), p38 inhibitor SB20190 (2.0 µM) or general ROS scavenger N-acetylcysteine (NAC) (5 mM).

### 2.7. Mitochondrial Reactive Oxygen Species Detection Assay

Mitochondrial ROS was measured with the use of MitoSox (Invitrogen, Carlsbad, CA, USA). Neutrophils were incubated with 5 µM of Mitosox in PBS prior to seeding in a 96-well plate. Incubation of neutrophils and read out was performed as described above. Experiments were done in duplicates, triplicates or quadruplicates depending on the number of neutrophils collected from the donor. For all conditions involving NETosis inhibitors, cells were preincubated either with DPI (20 μM), ERK-inhibitor FR180204 (20 µM), p38 inhibitor SB20190 (2.0 µM) or general ROS scavenger N-acetylcysteine (NAC) (5 mM).

### 2.8. Statistics

Data were analyzed by Mixed Models analysis, Friedman test or Wilcoxon test. As needed 2-WAY and 1-WAY ANOVA was applied with Bonferroni post-tests. A *p*-value of ≤0.05 was set as statistically significant. All statistical analyses were performed using GraphPad Prism version 5.03 (GraphPad Software, La Jolla, CA, USA) or SPSS (IBM, Amsterdam, The Netherlands). Data are expressed as mean +/− standard error of the mean (SEM). All experiments were done in threefold (*n* = 3, technical replicates, with ≥3 biological replicates unless stated).

## 3. Results

### 3.1. Stx2a Induces NET Formation in Healthy Human Neutrophils

**Figure 1:**
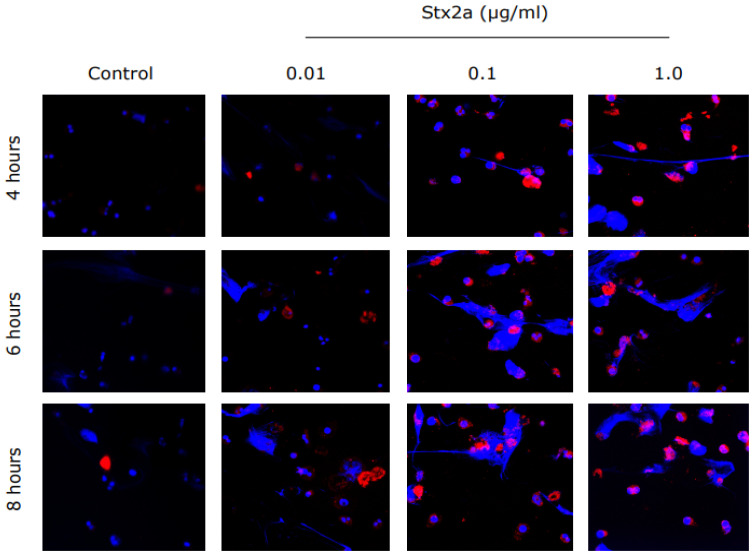
Stx2a-treated PMNs from healthy controls show expression of CD11b, an activation marker. Healthy control neutrophils were incubated with media (−ve control) or different concentrations of Stx2a over a period of 8 h. Immunostained neutrophils treated with Stx2a showed upregulated expression of CD11b in a concentration-dependent manner, and thus indicate activation of neutrophils. Images were captured with use of airy scan confocal microscopy at 63× magnification under oil immersion (*n* ≥ 3 and scale bar 20 µm).

**Figure 2:**
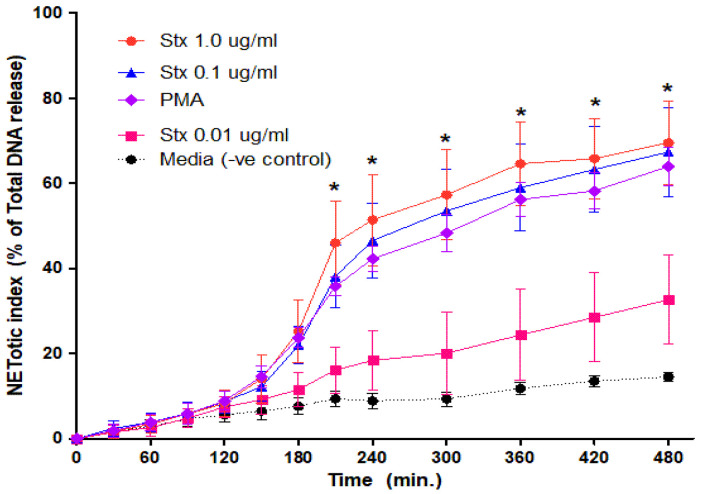
Dose-dependent NETs release response in neutrophils treated with Stx2a. Healthy control neutrophils were treated with different dosages of Stx2a, PMA, media (−ve control) and Triton (+ve control) and fluorescence reading was recorded up to 480 min. A dose-dependent effect was seen between Stx2a concentrations 0.01 µg/mL and 0.1 µg/mL, but not between 0.1 and 1.0 µg/mL of Stx2a. All significant differences were noted in conditions including 1.0 µg/mL, 0.1 µg/mL and PMA to media (−ve control) at indicated time points by a Mixed Models statistical test (* *p* < 0.05, *n* = 5).

To determine the effect of Stx2 on neutrophil activation and NET formation, we isolated neutrophils from healthy controls, incubated the cells with Stx2a and measured neutrophil activation marker CD11b. Image analyses showed that Stx2a directly upregulated the expression of CD11b on the cell surface of neutrophils in a concentration-dependent manner. CD11b was also diffusely stained in the cytosol of neutrophils. Images not only showed more immunofluorescence of CD11b (red), but also show a denser nucleus with DNA strings (blue) (Figure 1), which indicates cell activation or death [21]. The release of DNA was studied with the use of a SYTOX green assay. SYTOX green is a cell impermeable nucleic acid stain that binds DNA rapidly and with high affinity [22]. Neutrophils from healthy donors were incubated with different concentrations of Stx2a and the release of DNA was measured over a time period of 8 h. As shown in Figure 2, neutrophils released DNA after incubation with Stx2a in a time-dependent manner. A large amount of DNA release was observed when neutrophils were incubated with 0.1 µg/mL and 1.0 µg/mL of Stx2a, while this release was not significant compared to that of controls with 0.01 µg/mL Stx2a. These results show that Stx2a upregulates CD11b and induces NET formation in healthy human neutrophils, in a concentration-dependent manner.

### 3.2. Immunostaining Confirms NET Formation in Healthy Human Neutrophils by Stx2a

**Figure 3:**
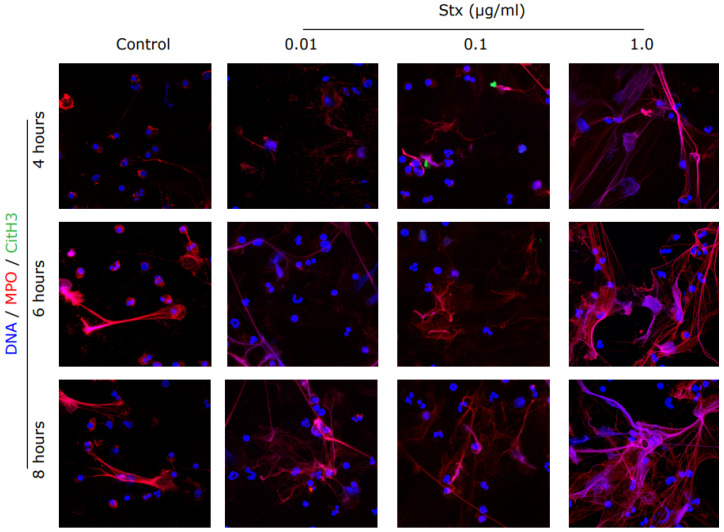
Immunostained images confirmed the release of NETs in Stx2a treated PMNs. Healthy control neutrophils were treated with media (−ve control) and different dosages of Stx2a. Cells were fixed and immunostained for citrullinated histone 3 (CitH3), myeloperoxidase (MPO) and DNA after 4, 6 and 8 h. NETs are visible as web-like structures positive for DNA (blue) decorated with myeloperoxidase (red). Staining of CitH3 (green) is not reported, indicating that Stx2a induces the Nox-dependent NETosis pathway (63× Magnification, *n* = 3 and scale bar 20 µm). For positive control and quantification, see Supplementary Figure S1.

To confirm the NET formation suggested by the SYTOX green assay, we immunostained and imaged the specimen. NETs not only consist of DNA, but also contain histones and granular proteins [23]. NETs should become visible as extra-cellular web-like structures positive for DNA in combination with one of its specific markers like CitH3 or MPO [23]. The presence of specific markers also depends on the pathways involved [17,24]. As shown by the images, neutrophils release NETs after stimulation with Stx2a (Figure 3). Images show the presence of DNA (blue) and MPO (red), while CitH3 (green) does not seem to be present (Figure 3; Supplementary Figure S1). MPO is a granular protein but coats the nuclear content and is subsequently released during NETosis. Microscopy showed that MPO (red) was located on the DNA strands (blue), as expected for NETs (Figure 3). CitH3 is mainly released after activation of neutrophils via the NOX-independent calcium-dependent pathway [17]; images with very little CitH3 staining suggest that NETs are released via the NOX-dependent pathway. Some NET are visible in media conditions after 6 and 8 h, which is expected to occur via spontaneous NETosis. Therefore, Stx2a dose-dependently induces NET formation in healthy human neutrophils.

### 3.3. Stx2a Induces NETosis via the NOX-Dependent Pathway

**Figure 4:**
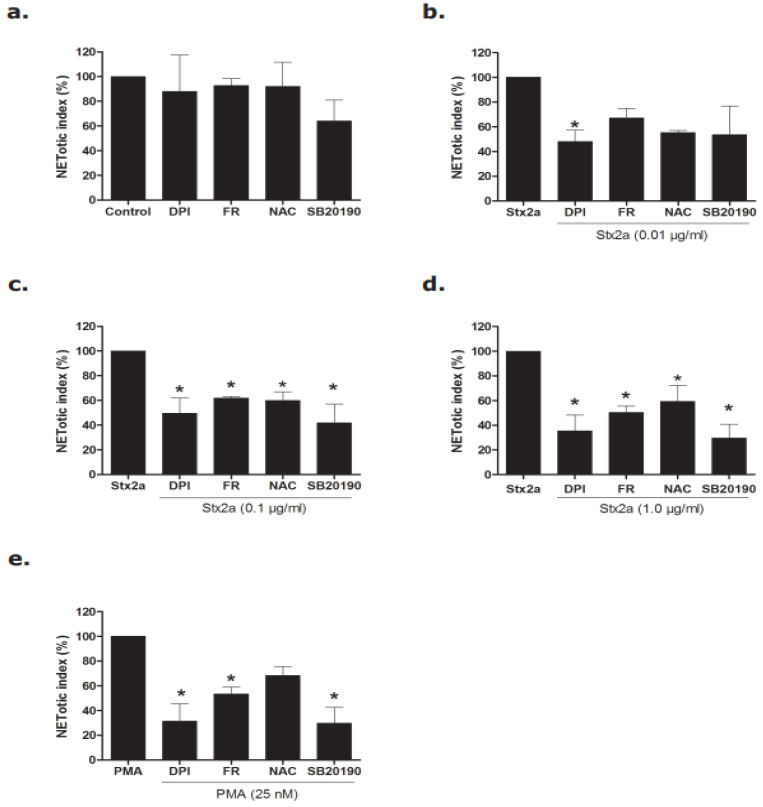
Inhibitors data confirmed that Stx2a induces NETosis via the NOX-dependent pathway. Neutrophils were treated with media (−ve cotrol), Stx, and PMA with and without different inhibitors (DPI, FR, NAC and SB20190) and a NETosis kinetics assay was performed. (**a**–**e**) Significant inhibition of NETs was found in neutrophils treated with Stx2a-at concentrations of 0.1 and 1.0 µg/mL after 4 h incubation when neutrophils were preincubated with FR, NAC and SB20190 (**b**,**c**). However, significant inhibition of DNA release was found for all Stx2a concentrations (0.01, 0.1 and 1.0 µg/mL) when neutrophils were preincubated with the NOX-specific inhibitor DPI (**b**–**d**). This strongly suggests a pivotal role of NOX in Stx2a-mediated NETosis, when considering a similar response of PMA-treated neutrophils (**e**) (* *p* < 0.05 compared to their respective control, 2-Way ANOVA with Bonferroni posttest, *n* = 4). For SytoxGreen plate images, and kinetics data, see supplementary Figures S2 and S3.

To confirm the pathways involved in Stx2a-induced NETosis, we used inhibitors specific for the various pathways involved in NETosis. Diphenyleneiodonium (DPI), a direct inhibitor of NOX [25], significantly inhibited Stx2a-induced NETosis at a concentration of 20 µM at all respective concentrations of Stx2a (Figure 4a–d; Supplementary Figures S2 and S3). This is consistent with the results that DPI significantly inhibits NOX-dependent NETosis of neutrophils induced by PMA (25 nM) (Figure 4e). With PMA being a generally accepted NOX-dependent agonist of NETosis, these findings suggest a pivotal role of NOX in Stx2a-induced NETosis. By contrast, ERK inhibition via FR180204 (20 µM), p38 inhibition via SB20190 (2.0 µM) or mitigated ROS signal transduction via N-acetylcysteine (NAC) (5 mM) were not able to significantly inhibit Stx2a-induced NETosis at the lowest concentration of Stx2a at 240 min (Figure 4b and Figure S3b), with significant inhibition occurring only at higher doses of Stx2a by 180 min (Figure 4c,d). These inhibitor studies show that Stx2a induces NETosis via the NOX-dependent pathway in a dose-dependent manner.

### 3.4. Stx2a-Activated Neutrophils Generate NOX-Mediated ROS

**Figure 5:**
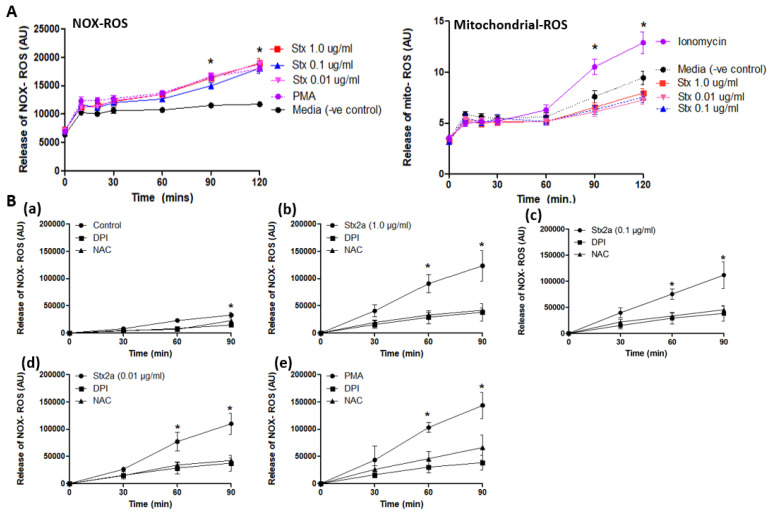
Stx2a induces NOX-ROS generation. Release of NOX-ROS and mitochondrial-ROS was measured by using DHR123, and MitoSox respectively. PMA and ionomycin were used as +ve control. (**A**) Data shows the involvement of NOX-ROS in Stx2a treated cells but not of mitochondrial ROS, there only +ve control (ionomycin) showed mitochondrial ROS generation (**B**) NOX-ROS release was measured along with DPI—a NOX-specific inhibitor, or NAC—a ROS scavenger. Untreated neutrophils (**a**) treated with 1.0–0.01 µg/mL of Stx2a (**b**–**d**) or 25 nM of PMA (**e**) with inhibitors over a time period of 90 min showed NOX-ROS generation. Significant differences were determined by a Mixed Models statistical test (*p* < 0.05). Significant differences were noted in conditions with their media control at respective time points (* *p* < 0.05 Stx2a compared to inhibitors DPI and NAC at respective time points, *n* = 4).

To further confirm the involvement of the NOX-pathway in *Stx2a*-mediated NETosis, the production of ROS was measured. ROS refers to a spectrum of radical and nonradical oxidants that could be generated by neutrophils [26,27]. The most relevant oxidants produced by neutrophils are those generated by MPO, an abundant peroxidase that uses hydrogen peroxide to oxidize substrates to reactive products [26,27]. In contrast to the NOX-independent pathway, the NOX-dependent pathway is highly dependent on the activation of ROS [17] and can be measured with DHR123, a ROS-sensitive dye. The NOX-independent pathway to release NETs is activated by influx of calcium [17] and mitochondrial ROS production. The release of mitochondrial ROS was measured with Mitosox, a mitochondrial specific ROS-sensitive dye. Healthy donor neutrophils were stimulated with different concentrations of Stx2a, and the release of general cytoplasmic ROS and mitochondrial ROS were measured (Figure 5A).

As shown in Figure 5, neutrophils released a large burst of ROS after incubation with Stx2a (Figure 5A). PMA, a protein kinase C agonist, is used as a positive control as it is known to stimulate the NOX-dependent ROS production [17]. However, it seems that mitochondrial ROS generation is not involved in neutrophils activated by Stx2a (Figure 5A). Ionomycin is used as positive control as it is known to cause a sufficient influx of calcium resulting in the stimulation of the NOX-independent pathway [17]. These results point towards the activation and involvement of the NOX-dependent pathway to release DNA, while the NOX-independent pathway does not seem to be directly activated by Stx2a. In addition, DPI (NOX specific inhibitor) and NAC (ROS scavenger) were used in combination with Stx2a or PMA to further show the role of NOX in Stx2a-induced NETosis (Figure 5B(a–e). Results demonstrated a similar inhibitory effect of DPI and NAC in neutrophils treated with Stx2a at various concentrations (Figure 5B(b–d)) and PMA (Figure 5B(e)), a classical NOX-dependent agonist, thus confirming the role of NOX in ROS production in Stx2a-induced NETosis.

### 3.5. Neutrophils from STEC-HUS Patients Are More Prone to Undergo Spontaneous NETosis

**Figure 6:**
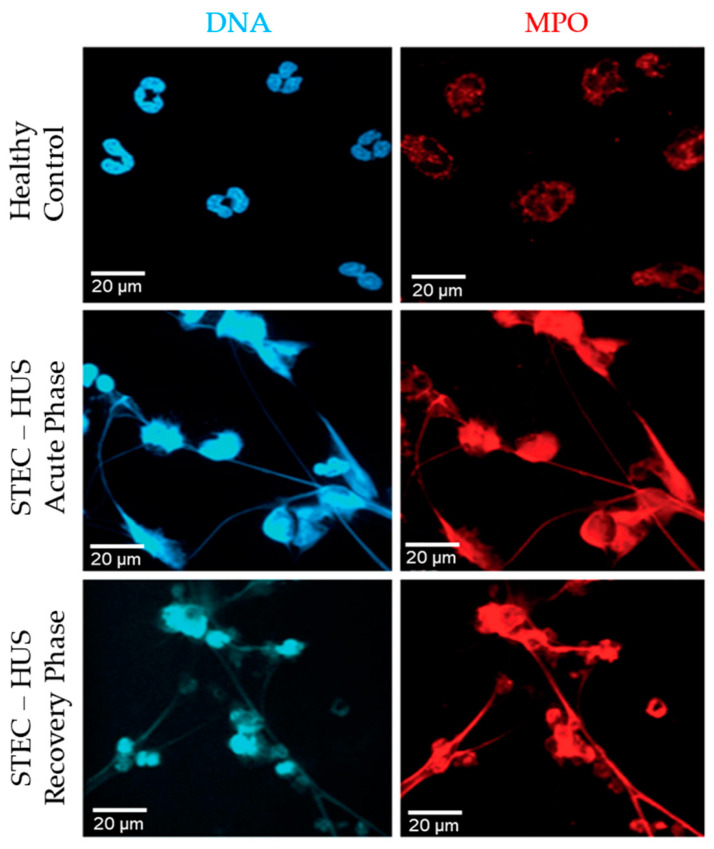
Spontaneous NETosis of neutrophils derived from patients with STEC-HUS. Immunostained images after 4 h of incubation show spontaneous release of NETs by neutrophils derived from patients in acute and recovery phase of disease, while this effect is not seen in healthy control neutrophils. Web-like structures representing NETs are positively stained for DNA (blue) and MPO (red). 63× magnification, *n* = 3 and scale bar 20 µm.

We hypothesized that neutrophils from STEC-HUS patients behave differently from healthy donor neutrophils in terms of the release of NETs and the pathways involved. In this study, neutrophils from three pediatric patients with STEC-HUS in the acute phase of disease and after 3–5 months of recovery were isolated (Table 1). All three patients were of the age of 8 years with an unknown source of STEC-infection. The neutrophil count of each patient in the acute phase of disease was respectively 6 × 10^9^/L, 10.6 × 10^9^/L and 10.5 × 10^9^/L, while the neutrophil count during discharge was decreased till 5.2 × 10^9^/L, 7.2 × 10^9^/L and 7.9 × 10^9^/L. No neutrophil count was measured after 3–5 months of recovery. In media conditions (−ve control, no agonist), neutrophils from patients in both the acute and recovery phase of disease showed the spontaneous release of NETs (Figure 6). This effect was not seen in healthy control neutrophils. Second, healthy control neutrophils retained there typical multilobular nucleus, while STEC-HUS patient neutrophils showed a denser and delobular nucleus (Figure 6).

Thirdly, the web-like structures seen in the images represent the NETs. Those structures are highly positive for DNA (blue) and MPO (red). Those results show that neutrophils from STEC-HUS patients are more prone to undergo spontaneous NETosis and that this spontaneous NETosis is activated by the NOX-dependent pathway.

### 3.6. NOX-Dependent Pathway in the Acute Phase of STEC-HUS Is Less Activated Ex Vivo

**Figure 7:**
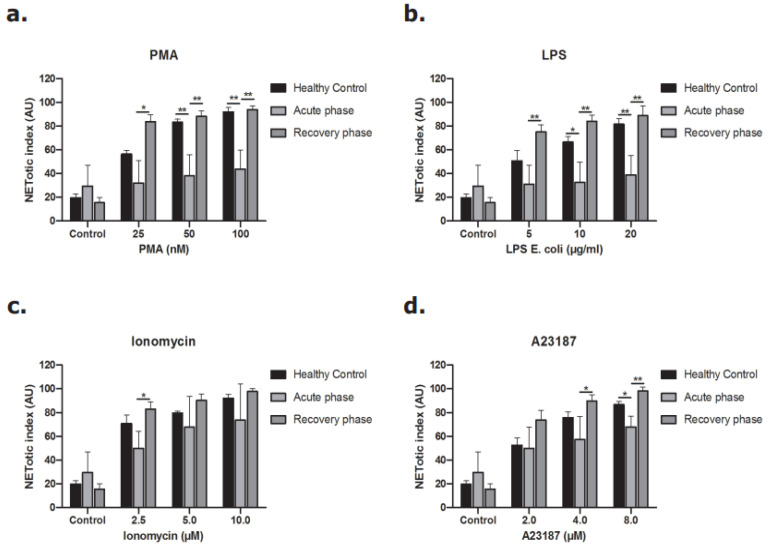
Release of DNA from neutrophils of STEC-HUS patients. Neutrophils derived from healthy donors and STEC-HUS patients in the acute phase and recovery phase of disease were incubated with (**a**) PMA, (**b**) LPS, (**c**) A23187 and (**d**) Ionomycin for 4 h to stimulate NETs release. Data show less release of NETs by neutrophils in the acute phase of disease in both the NOX-dependent pathway and also in the NOX-independent pathway but with lesser extent. (* *p* ≤ 0.05, ** *p* ≤ 0.01, *n* > 3, Friedman test and Wilcoxon test were applied).

Neutrophils were incubated with different concentrations of PMA and LPS to stimulate the NOX-dependent pathway and different concentrations of ionomycin and A23187 to study the NOX-independent pathway [13,28,29]. As shown in Figure 7, neutrophils in the acute phase of disease released less DNA after stimulation of the NOX-dependent pathway (Figure 7a,b). The same pattern was observed when the NOX-independent pathway was activated, but to a lesser extent (Figure 7c,d). The release of NETs during the recovery phase of disease was re-established for both the NOX-dependent and the NOX-independent pathway.

**Figure 8:**
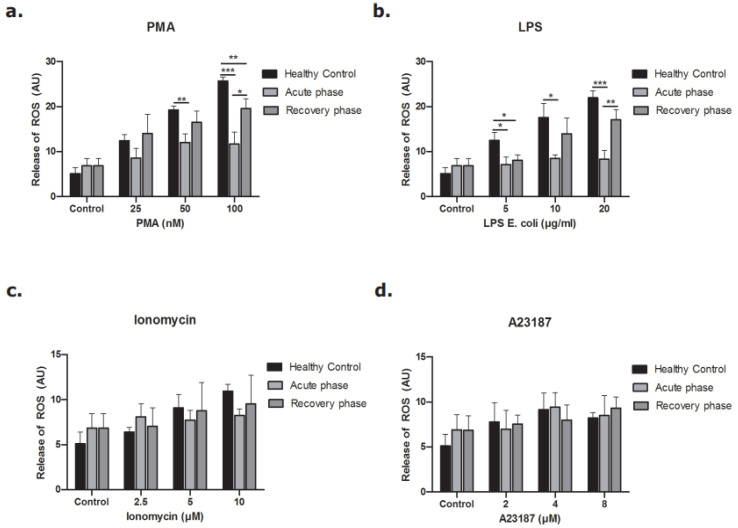
Release of ROS from neutrophils of STEC-HUS patients. Neutrophils derived from healthy donors and STEC-HUS patients in the acute phase and recovery phase of disease were incubated with (**a**) PMA, (**b**) LPS, (**c**) Ionomycin, (**d**) A23187 for 4 h to estimate ROS generated by NOX-dependent and -independent pathways. Data show less generation of ROS by neutrophils in the acute phase of disease when the NOX-dependent pathway is activated. The respective significant comparisons are indicated by horizontal bar with (* *p* ≤ 0.05, ** *p* ≤ 0.01, *** *p* ≤ 0.005, *n* = 3, Friedman test and Wilcoxon test were applied).

When we studied the production of ROS, similar results were seen. ROS production by the NOX-dependent pathway was less in neutrophils derived during the acute phase of STEC-HUS (Figure 8a,b), while there was no clear difference after stimulation of the NOX-independent pathway (Figure 8c,d). ROS production by activation of the NOX-dependent pathway was partly re-established during the recovery phase of disease. Again, no difference was found after stimulation of the NOX-independent pathway. Altogether, those results demonstrate that neutrophils in the acute phase of STEC-HUS release less DNA and less ROS by the NOX-dependent pathway ex vivo compared to that of neutrophils in the recovery phase of disease and healthy control neutrophils.

## 4. Discussion

In this paper, we demonstrate that Stx2a is able to directly activate neutrophils. Stx2a activated neutrophils release NETs via the NOX-dependent pathway with the production of ROS, while the NOX-independent pathway does not seem to be involved as indicated by the absence of substantial mitochondrial-specific ROS production and citrullinated H3 formation. Secondly, the behavior of neutrophils from healthy donors and neutrophils isolated from patients with STEC-HUS in the acute and recovery phase of disease was studied. Neutrophils from STEC-HUS patients in the acute phase are more prone to undergo spontaneous NETosis by the NOX-dependent pathway, and this effect was still present during the recovery phase of disease. Thirdly, unexpectedly neutrophils from patients with STEC-HUS in the acute phase of disease showed less NETs release and ROS production by the NOX-dependent pathway likely due to a state of exhaustion.

Brigotti et al. (2010) and Ramos et al. (2016) both showed the direct consequences of Stx on neutrophils with upregulation of different activation markers, degranulation, and the release of NETs [8,10]. We were interested in identifying the underlying pathways involved and showed that this activation is mainly caused by stimulation of the NOX-dependent pathway. In contrast to the studies mentioned above and our own data, Fernandez et al. (2005) did not find any effect of Stx on the expression of CD11b, CD16, CD66b and MPO even though they used approximately 100 times higher concentrations of Stx than we used in this study [9]. Priming of neutrophils with TNF-alpha, IL-8 or LPS did not modify their results. One of the main explanations could be a difference of effect dependent on the type and structure of Stx used and therefore different binding characteristics of Stx. Brigotti et al. (2019) published different binding abilities of Stx to neutrophils dependent on the purification and the structure of the toxin [30]. Even though we didn’t study the binding capacity of Stx, the toxin we used was able to activate neutrophils, so the structure of the toxin might be comparable with the uncleaved toxin able to bind to neutrophils used in their paper.

NETosis is a complex process and the molecular events driving NETosis substantially overlap between the NOX-dependent and NOX-independent pathways [17]. While these two pathways indeed show hallmarks of distinction, they should not be considered mutually exclusive. Using specific inhibitors, we dissected the contribution of these two pathways in Stx2a induced NETosis. DPI is a known specific inhibitor of NOX, and in matching previous studies, we demonstrated that DPI effectively inhibited PMA-induced NETosis, as expected given that PMA is a confirmed canonical NOX-dependent NETosis agonist. Our findings show that DPI inhibits both ROS and NETs release in Stx2a-stimulated neutrophils and similarly also in PMA-treated control neutrophils. Our results are in support of a pivotal role of NOX in Stx2a-induced NETosis. This is in keeping with our experimental results demonstrating that NAC significantly inhibits both ROS activity and the resulting NETosis of control neutrophils treated with Stx2a. Further supporting the central role for NOX in Stx2a-induced NETosis, downstream pathway inhibitors FR180204 (ERK inhibitor) and SB20190 (p38 inhibitor) inhibit Stx2a- and PMA-induced NETosis similarly.

Our data are in line with Ramos et al. (2016) who showed for the first time that neutrophils from STEC-HUS patients are more prone to undergo spontaneous NETosis [10]. We confirmed this in the acute phase of STEC-HUS. Surprisingly, spontaneous NETosis was still visible in the recovery phase of the disease while this was not the case for healthy donor neutrophils. We isolated neutrophils from the same donors after 3–5 months of recovery. This period of time should be long enough for neutrophils to recover after the start of infection as neutrophils have a circulating half-life of 6 till 8 h [31]. However, systemic inflammatory alterations that affect the response of neutrophils could still be present after several months of disease. It would be good to study the spontaneous NETosis of neutrophils derived from patients after 1 year of recovery from STEC-HUS to see if spontaneous NETosis still occurs, especially in a larger cohort. It might be the case that neutrophils from STEC-HUS patients in general show more NETs generation independent from the phase of disease they are in, for example, caused by epigenetic differences. This phenomenon applies to various inflammatory and auto-immune diseases like rheumatoid arthritis and systemic lupus erythematosus [32]. It would be interesting to study the release of NETs under the influence of sera from STEC-HUS patients in the acute and recovery phase of disease to see if enhanced NET formation is stimulated by certain factors like cytokines and humoral factors present in the serum. Leffler et al. (2017) published the decreased ability to degrade NETs in patients with STEC-HUS during both the acute phase of disease and after remission (mean remission of 38 days after the acute phase) [7]. Their group showed that this was associated with decreased nuclease activity. Interestingly, impaired degradation of NETs caused by autoantibodies against double-stranded DNA that block the access of nucleases was found in lupus nephritis [7].

In the past, Fernandez et al. (2005) stimulated healthy donor neutrophils and neutrophils from patient in the acute and recovery phase of STEC-HUS with PMA and measured ROS with the use of DHR in combination with flow cytometry. They showed less PMA-triggered ROS generation by neutrophils in the acute phase of disease and showed that this effect was partly restored in the recovery phase, which is in line with our results [9]. In our study, we added LPS (NOX-dependent), ionomycin and A23187 (both NOX-independent) to distinguish between the NOX-dependent and NOX-independent pathway. With the use of those agonists, we were able to show that general ROS production of neutrophils (NOX-dependent pathway) was decreased in the acute phase of disease, but that mitochondrial ROS production (NOX-independent pathway) was still intact. In contrast to the ROS production, the NETotic index did show a significant difference after stimulation of the NOX-independent pathway when 8.0 µM of A23187 was used. This effect was less than the effect seen after stimulation of the NOX-dependent pathway and was not present when the NOX-independent pathway was activated by ionomycin. This might point towards another pathway or player involved in the release of DNA as the levels of ROS production were similar. However, the same result would be expected for ionomycin, as both are calcium agonists.

During infection, not only Stx is involved, but also different inflammatory cytokines and many of them are able to trigger neutrophils to release NETs, examples are LPS and TNF-alpha [33]. Both LPS and TNF-alpha also play a role in the pathophysiology of STEC-HUS [34]. Another system active during periods of infection is the complement system, a system part of the innate immunity [35,36]. Recently, it was published that complement activation directly activates neutrophils to release NETs [37,38] and complement is activated in STEC-HUS patients [39]. The interaction between neutrophils and platelets is also published with clot formation as result [25,39,40]. Endothelial damage with thrombus formation is the main cause of kidney failure in patients with STEC-HUS thus more information about the interaction between Stx, neutrophils and platelets in combination with glomerular endothelium would be of high interest. Future coculture experiments may allow us to gain new insights into those different interactions.

To the best of our knowledge, this is the first study that shows the activation of the NOX-dependent pathway by Stx2a and the difference in activation between the NOX-dependent and NOX-independent pathways in patients with STEC-HUS. The spontaneous NET formation that still happens in the recovery phase of STEC-HUS needs further investigation with increased sample size.

## Figures and Tables

**Table 1:** Clinical and laboratory features of STEC-HUS patients used in this study.

	Patient 1 Acute	After 5 Months Recovery	Patient 2 Acute	After 3 Months Recovery	Patient 3 Acute	After 3 Months Recovery
**Gender (M/F)**	M		F		M	
**Age (years)**	8		8		8	
***E. coli serotype***	Non-O157		O157		O157	
**Shiga toxin type**	Stx2		GI path negative		Stx2	
**Neutrophils** **(1.5–9.0 × 10^9^/L)**	6.0	N/A	10.6	N/A	10.5	N/A
**Leucocytes** **(4.0–10.0 × 10^9^/L)**	9.0	6.7	16.8	5.2	13.8	6.7
**Platelets** **(150–400 × 10^9^/L)**	78	348	92	277	30	198
**Hemoglobin in g/L** **(106–132 g/L)**	95	131	96	109	78	122
**LDH** **(134–225 U/L)**	5563	N/A	6392	N/A	8238	N/A
**Creatinine in µmol/L** **(M = < 80–125 µmol/L** **V = < 70–100 µmol/L)**	204	34	341	39	220	35
**Need for dialysis**	No		Yes		No	

## Data Availability

No associated data is available.

## Supplementary Materials

The following are available online at https://www.mdpi.com/article/10.3390/biomedicines9121807/s1, Figure S1: Stx2a mediated NETosis does not require H3 citrullination (CitH3). Figure S2: Sytox-Green plate images confirmed the NETosis and NETs suppression by various inhibitors. Figure S3: Kinetics of the NETosis inhibition under different stimulants.

## References

[1] George J.N., Nester C.M. (2014). Syndromes of thrombotic microangiopathy. N. Engl. J. Med..

[2] Tarr P.I., Gordon C.A., Chandler W.L. (2005). Shiga-toxin-producing Escherichia coli and haemolytic uraemic syndrome. Lancet.

[3] Karmali M.A. (2004). Infection by Shiga toxin-producing Escherichia coli. Mol. Biotechnol..

[4] Mele C., Remuzzi G., Noris M. (2014). Hemolytic uremic syndrome. Seminars in Immunopathology.

[5] Rosales A., Hofer J., Zimmerhackl L.-B., Jungraithmayr T.C., Riedl M., Giner T., Strasak A., Orth-Höller D., Würzner R., Karch H. (2012). Need for long-term follow-up in enterohemorrhagic Escherichia coli–associated hemolytic uremic syndrome due to late-emerging sequelae. Clin. Infect. Dis..

[6] Petruzziello T.N., Mawji I.A., Khan M., Marsden P.A. (2009). Verotoxin biology: Molecular events in vascular endothelial injury. Kidney Int..

[7] Leffler J., Prohászka Z., Mikes B., Sinkovits G., Ciacma K., Farkas P., Réti M., Kelen K., Reusz G.S., Szabó A.J. (2017). Decreased neutrophil extracellular trap degradation in Shiga toxin-associated haemolytic uraemic syndrome. J. Innate Immun..

[8] Brigotti M., Tazzari P.L., Ravanelli E., Carnicelli D., Barbieri S., Rocchi L., Arfilli V., Scavia G., Ricci F., Bontadini A. (2010). Endothelial damage induced by Shiga toxins delivered by neutrophils during transmigration. J. Leukoc. Biol..

[9] Fernández G.C., Gómez S.A., Rubel C.J., Bentancor L.V., Barrionuevo P., Alduncín M., Grimoldi I., Exeni R., Isturiz M.A., Palermo M.S. (2005). Impaired neutrophils in children with the typical form of hemolytic uremic syndrome. Pediatric Nephrol..

[10] Ramos M.V., Mejias M.P., Sabbione F., Fernandez-Brando R.J., Santiago A.P., Amaral M.M., Exeni R., Trevani A.S., Palermo M.S. (2016). Induction of neutrophil extracellular traps in Shiga toxin-associated hemolytic uremic syndrome. J. Innate Immun..

[11] Brinkmann V., Zychlinsky A. (2007). Beneficial suicide: Why neutrophils die to make NETs. Nat. Rev. Microbiol..

[12] Milford D., Staten J., MacGreggor I., Dawes J., Taylor C., Hill F. (1991). Prognostic markers in diarrhoea-associated haemolytic-uraemic syndrome: Initial neutrophil count, human neutrophil elastase and von Willebrand factor antigen. Nephrol. Dial. Transplant..

[13] Brinkmann V., Reichard U., Goosmann C., Fauler B., Uhlemann Y., Weiss D.S., Weinrauch Y., Zychlinsky A. (2004). Neutrophil extracellular traps kill bacteria. Science.

[14] Khan M.A., Farahvash A., Douda D.N., Licht J.-C., Grasemann H., Sweezey N., Palaniyar N. (2017). JNK Activation Turns on LPS-and Gram-Negative Bacteria-Induced NADPH Oxidase-Dependent Suicidal NETosis. Sci. Rep..

[15] Douda D.N., Yip L., Khan M.A., Grasemann H., Palaniyar N. (2014). Akt is essential to induce NADPH-dependent NETosis and to switch the neutrophil death to apoptosis. Blood.

[16] Azzouz D., Khan M.A., Palaniyar N. (2021). ROS induces NETosis by oxidizing DNA and initiating DNA repair. Cell Death Discov..

[17] Khan M.A., Palaniyar N. (2017). Transcriptional firing helps to drive NETosis. Sci. Rep..

[18] Joseph A., Cointe A., Mariani Kurkdjian P., Rafat C., Hertig A. (2020). Shiga toxin-associated hemolytic uremic syndrome: A narrative review. Toxins.

[19] Naffah de Souza C., Breda L.C., Khan M.A., Almeida S.R.d., Câmara N.O.S., Sweezey N., Palaniyar N. (2018). Alkaline pH promotes NADPH oxidase-independent neutrophil extracellular trap formation: A matter of mitochondrial reactive oxygen species generation and citrullination and cleavage of histone. Front. Immunol..

[20] Azzouz D., Khan M.A., Sweezey N., Palaniyar N. (2018). Two-in-one: UV radiation simultaneously induces apoptosis and NETosis. Cell Death Discov..

[21] Elmore S. (2007). Apoptosis: A review of programmed cell death. Toxicol. Pathol..

[22] Thakur S., Cattoni D.I., Nöllmann M. (2015). The fluorescence properties and binding mechanism of SYTOX green, a bright, low photo-damage DNA intercalating agent. Eur. Biophys. J..

[23] Brinkmann V., Goosmann C., Kühn L.I., Zychlinsky A. (2013). Automatic quantification of in vitro NET formation. Front. Immunol..

[24] Pieterse E., Rother N., Yanginlar C., Gerretsen J., Boeltz S., Munoz L.E., Herrmann M., Pickkers P., Hilbrands L.B., van der Vlag J. (2018). Cleaved N-terminal histone tails distinguish between NADPH oxidase (NOX)-dependent and NOX-independent pathways of neutrophil extracellular trap formation. Ann. Rheum. Dis..

[25] Ståhl A.-l., Sartz L., Nelsson A., Békássy Z.D., Karpman D. (2009). Shiga toxin and lipopolysaccharide induce platelet-leukocyte aggregates and tissue factor release, a thrombotic mechanism in hemolytic uremic syndrome. PLoS ONE.

[26] Winterbourn C.C., Kettle A.J., Hampton M.B. (2016). Reactive oxygen species and neutrophil function. Annu. Rev. Biochem..

[27] Aratani Y. (2018). Myeloperoxidase: Its role for host defense, inflammation, and neutrophil function. Arch. Biochem. Biophys..

[28] Fuchs T.A., Brill A., Duerschmied D., Schatzberg D., Monestier M., Myers D.D., Wrobleski S.K., Wakefield T.W., Hartwig J.H., Wagner D.D. (2010). Extracellular DNA traps promote thrombosis. Proc. Natl. Acad. Sci. USA.

[29] Ravindran M., Khan M.A., Palaniyar N. (2019). Neutrophil Extracellular Trap Formation: Physiology, Pathology, and Pharmacology. Biomolecules.

[30] Brigotti M., Orth-Höller D., Carnicelli D., Porcellini E., Galassi E., Tazzari P.L., Ricci F., Manoli F., Manet I., Talasz H. (2019). The structure of the Shiga toxin 2a A-subunit dictates the interactions of the toxin with blood components. Cell. Microbiol..

[31] Summers C., Rankin S.M., Condliffe A.M., Singh N., Peters A.M., Chilvers E.R. (2010). Neutrophil kinetics in health and disease. Trends Immunol..

[32] Gupta S., Kaplan M.J. (2016). The role of neutrophils and NETosis in autoimmune and renal diseases. Nat. Rev. Nephrol..

[33] Van Avondt K., Hartl D. (2018). Mechanisms and disease relevance of neutrophil extracellular trap formation. Eur. J. Clin. Investig..

[34] Van de Kar N., Monnens L., Karmali M.A., van Hinsbergh V. (1992). Tumor necrosis factor and interleukin-1 induce expression of the verocytotoxin receptor globotriaosylceramide on human endothelial cells: Implications for the pathogenesis of the hemolytic uremic syndrome. Blood.

[35] Walport M.J. (2001). Complement. N. Engl. J. Med..

[36] Walport M.J. (2001). Complement. Second of two parts. N. Engl. J. Med..

[37] Guglietta S., Chiavelli A., Zagato E., Krieg C., Gandini S., Ravenda P.S., Bazolli B., Lu B., Penna G., Rescigno M. (2016). Coagulation induced by C3aR-dependent NETosis drives protumorigenic neutrophils during small intestinal tumorigenesis. Nat. Commun..

[38] Yuen J., Pluthero F.G., Douda D.N., Riedl M., Cherry A., Ulanova M., Kahr W.H., Palaniyar N., Licht C. (2016). NETosing neutrophils activate complement both on their own NETs and bacteria via alternative and non-alternative pathways. Front. Immunol..

[39] Westra D., Volokhina E.B., van der Molen R.G., van der Velden T.J., Jeronimus-Klaasen A., Goertz J., Gracchi V., Dorresteijn E.M., Bouts A.H., Keijzer-Veen M.G. (2017). Serological and genetic complement alterations in infection-induced and complement-mediated hemolytic uremic syndrome. Pediatric Nephrol..

[40] Clark S.R., Ma A.C., Tavener S.A., McDonald B., Goodarzi Z., Kelly M.M., Patel K.D., Chakrabarti S., McAvoy E., Sinclair G.D. (2007). Platelet TLR4 activates neutrophil extracellular traps to ensnare bacteria in septic blood. Nat. Med..

