# Supplementary material for: Shiga Toxin 2a Induces NETosis via NOX-Dependent Pathway
Source: Biomedicines. 2021 Dec 1;9(12):1807. doi: 10.3390/biomedicines9121807 (PMC8698832; doi:10.3390/biomedicines9121807)
Supplement: Supplementary file 1 [file biomedicines-09-01807-s001.zip › biomedicines-1455315-supplementary.pdf]

biomedicines

MDPI

Article

Shiga Toxin 2a Induces NETosis via NOX-Dependent Pathway

Wouter J. C. Feitz [1,2,†], Samuel Suntharalingham [2,3,†], Meraj Khan [3], Carolina G. Ortiz-Sandoval [2], Nades Palaniyar [3,4], Lambert P. van den Heuvel [1,5,6], Nicole C. A. J. van de Kar [1] and Christoph Licht [2,4,7,8,*]

[1] Department of Pediatric Nephrology, Amalia Children's Hospital, Radboud Institute for Molecular Life Sciences, Radboudumc, 6525 GA Nijmegen, The Netherlands; wouter.feitz@radboudumc.nl (W.J.C.F.); bert.vandenheuvel@radboudumc.nl (L.P.v.d.H.); nicole.vandekar@radboudumc.nl (N.C.A.J.v.d.K.)
[2] Cell Biology Program, The Hospital for Sick Children Research Institute, Toronto, ON M5G 1X8, Canada; samuel.elijah.suntharalingham@gmail.com (S.S.); carolina.ortiz@sickkids.ca (C.G.O.-S.)
[3] Program in Translational Medicine, The Hospital for Sick Children Research Institute, Toronto, ON M5G 1X8, Canada; meraj.khan@sickkids.ca (M.K.); nades.palaniyar@sickkids.ca (N.P.)
[4] Department of Laboratory Medicine and Pathobiology, and Institute of Medical Sciences, University of Toronto, Toronto, ON M5S1A8, Canada
[5] Department of Pediatric Nephrology, Academic Hospitals Leuven, 3000 Leuven, Belgium
[6] Department of Development and Regeneration, Academic Hospitals Leuven, 3000 Leuven, Belgium
[7] Division of Nephrology, The Hospital for Sick Children, Toronto, ON M5G 1X8, Canada
[8] Department of Pediatrics, University of Toronto, Toronto, ON M5S1A8, Canada
* Correspondence: christoph.licht@sickkids.ca
† Both authors contributed equally to the manuscript.

Citation: Feitz, W.J.C.; Suntharalingham, S.; Khan, M.; Ortiz-Sandoval, C.G.; Palaniyar, N.; van den Heuvel, L.P.; van de Kar, N.C.A.J.; Licht, C. Shiga Toxin 2a Induces NETosis via NOX-Dependent Pathway. *Biomedicines* **2021**, *9*, 1807. https://doi.org/10.3390/biomedicines9121807

Academic Editor: Shaker A. Mousa

Received: 25 October 2021
Accepted: 25 November 2021
Published: 1 December 2021

Publisher's Note: MDPI stays neutral with regard to jurisdictional claims in published maps and institutional affiliations.

Copyright: © 2021 by the authors. Licensee MDPI, Basel, Switzerland. This article is an open access article distributed under the terms and conditions of the Creative Commons Attribution (CC BY) license (http://creativecommons.org/licenses/by/4.0/).

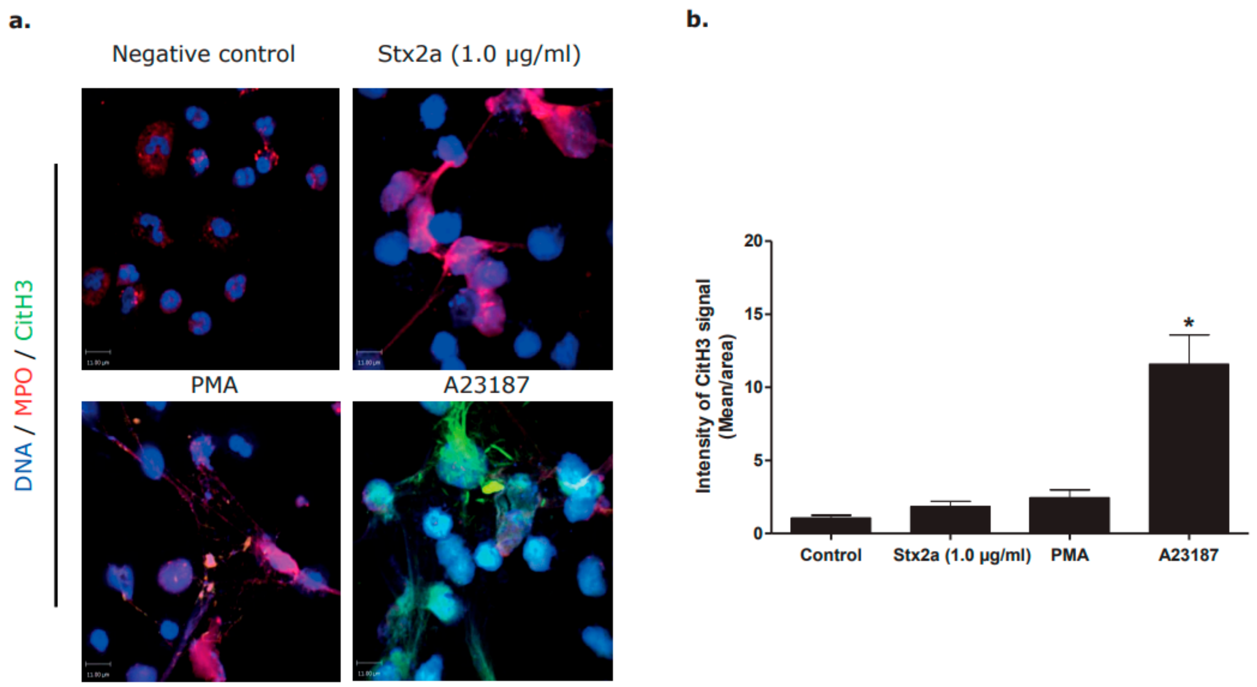

Figure S1. Stx2a mediated NETosis does not require H3 citrullination (CitH3). Control neutrophils were incubated with either media (negative control), Stx2a (1.0 ug/mL), PMA (25 nM) or calcium ionophore A23187 (4 µM) for 4 hours. (**a**) Cells were fixed and stained for DNA, citrullinated histone 3 and myeloperoxidase after 4 hours. NETs are visible as web-like structures positive for DNA (blue) and myeloperoxidase (red). While media (negative control), Stx2a (1.0 ug/mL) and PMA incubated neutrophils stained negative for citrullinated H3 (green), only neutrophils incubated with A23187 stained positive. These results are in line with data shown in Figure 2 and further support that Stx2a induces NETosis via the NOX-dependent pathway. In addition, these results suggest the absence of a substantial calcium influx during Stx2a-induced NETosis. (**b**) CitH3 was quantified via Image J using a total of 5 visual fields per condition for all biological replicates. Images were recorded via an Olympus IX81 (Olympus corporation, Tokyo, Japan) inverted fluorescence microscope at 40X. The figure shows representative images. (* $p < 0.05$, n > 3, One-way ANOVA Bonferroni post-hoc test was used to determine significance and scale bar 11 µm).

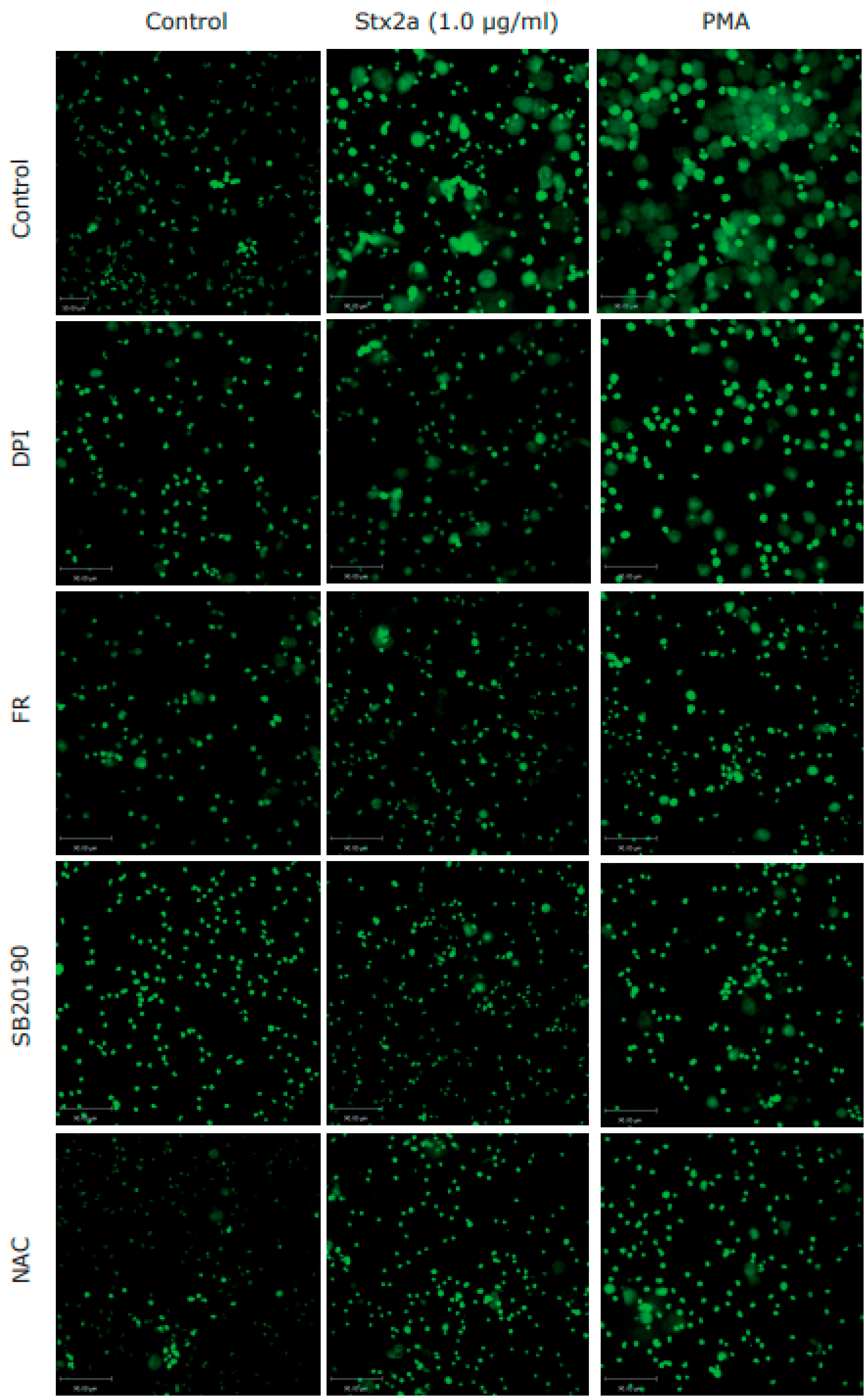

Figure S2. Sytox-Green plate images confirmed the NETosis and NETs suppression by various inhibitors. To estimate NETosis kinetics of neutrophils, a SYTOX green fluorescence assay was used. A concentration of 5 µM SYTOX green in 100 ul media containing 50.000 neutrophils was added. This was seeded into a 96-well plate. Neutrophils were either left untreated (control) or were treated with Stx2a (1.0 µg/mL) or PMA (25 nM) for 4 hours. Following the SYTOX green fluorescence assay, images were recorded via an Olympus IX81 (Olympus corporation, Tokyo, Japan) inverted fluorescence microscope at 20X. (n = 3, and scale bar 90 µm).

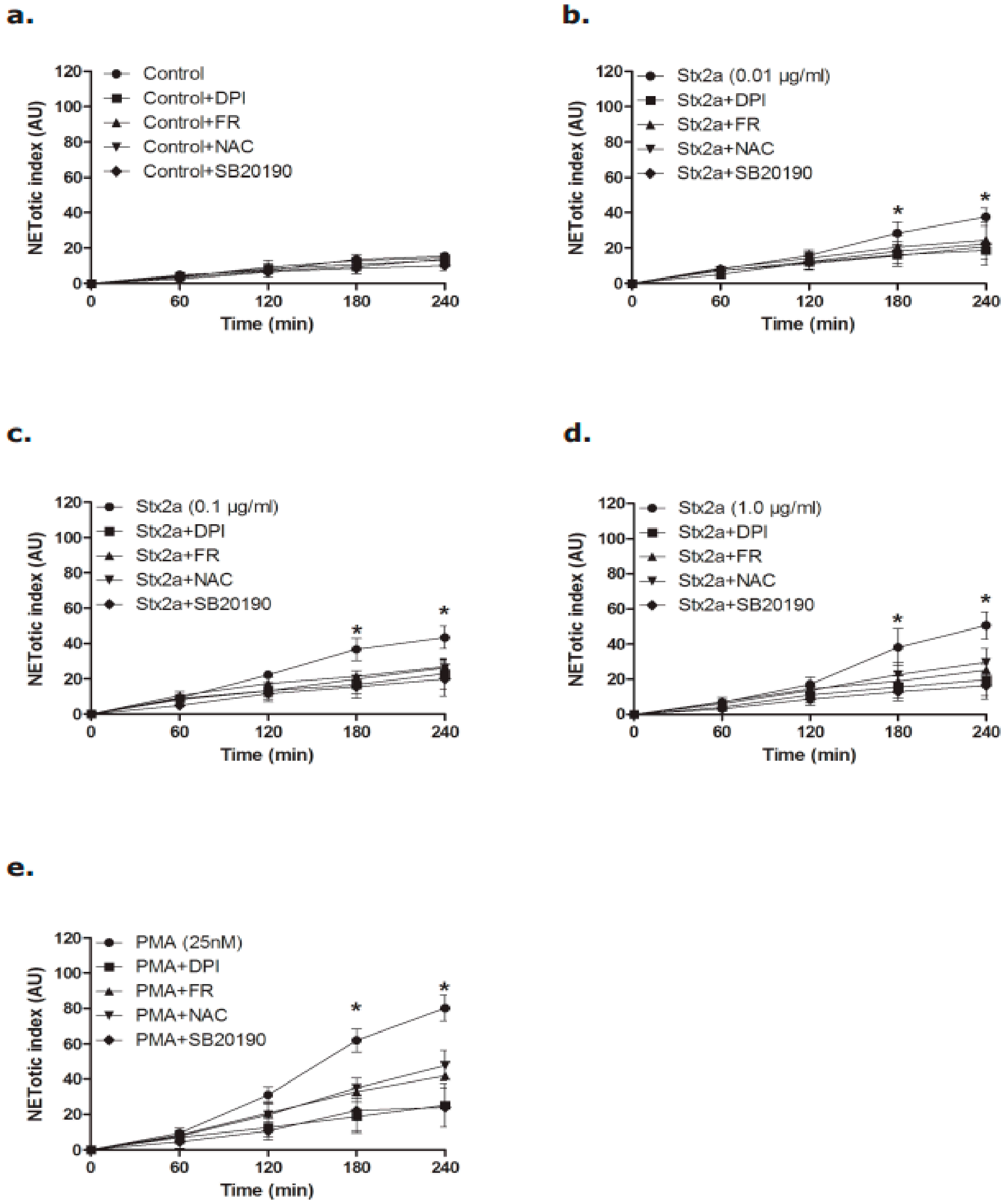

Figure S3. Kinetics of the NETosis inhibition under different stimulants. These graphs show the kinetics of the NETosis (%DNA release at each time under different conditions. The detail legend and descriptions are available in Figure 4.
